# Procedural Outcome and Long-Term Follow-Up of Patients Undergoing Epicardial or Endocardial Left Atrial Appendage Occlusion

**DOI:** 10.3390/jcm15114275

**Published:** 2026-06-01

**Authors:** Karin Nentwich, Nuki Kaziashvilli, Elena Sauer, Artur Berkovitz, Julian Mueller, Anja Schade, Sebastian Barth, Ulrich Luesebrink, Thomas Deneke

**Affiliations:** 1 Department of Invasive Electrophysiology, Campus Bad Neustadt, Von Guttenbergstrasse 1, 97616 Bad Neustadt/Saale, Germany; nuki.kaziashvilli@campus-nes.de (N.K.); sauer.elena@campus-nes.de (E.S.); artur.berkovitz@campus-nes.de (A.B.); anja.schade@campus-nes.de (A.S.); 2 Department of Cardiology, Phillips-University of Marburg, 35043 Marburg, Germany; sebastian.barth@campus-nes.de (S.B.); ulrich.luesebrink@uk-gm.de (U.L.); 3 Department of Cardiology, Universitäts-Herzzentrum Bad Krozingen, 79189 Bad Krozingen, Germany; julian.mueller@uniklinik-freiburg.de; 4 Department of Cardiology and Imaging, Campus Bad Neustadt, 97616 Bad Neustadt/Saale, Germany; 5 Department of Cardiology, Clinic Nuernberg Campus South, 90471 Nuernberg, Germany; thomas.deneke@rub.de

**Keywords:** atrial fibrillation, contraindication for anticoagulation, left atrial appendage closure

## Abstract

**Introduction**: In high-risk atrial fibrillation patients, the endocardial LAA closure technique is the most common approach. Epicardial ligation is not as widely spread despite the advantage of omitting all anticoagulation straight after ligation. This study retrospectively analyzes endo- and epicardial LAA occlusion techniques in regard to efficacy, safety and long-term data. **Method**: From November 2018 to August 2024, 112 patients underwent LAA occlusion, 69 with an epicardial approach using Lariat^®^ (epicardial group) and 43 patients with an endocardial plug Amulet^TM^ (endocardial group). **Results**: A total of 69 patients were treated with epicardial ligation and 43 patients with implantation of an endocardial plug. Procedure time (mean of 83 min vs. 62 min) and fluoroscopy time (17 min vs. 6.16 min) were significantly longer in the epicardial ligation group. Silent cerebral lesions (SCLs) in MRI were equally distributed in both groups (11.0% vs. 8%, *p* = 0.67). A 3-month FUP revealed five thrombi at the closure site in the epicardial group, which resolved with OAC therapy, and 0 in the endocardial group (8% vs. 0%), one central gap in the epicardial group and six peridevice leaks (PDLs) in the endocardial group (1% vs. 16%). A 12-month FUP revealed no thrombus in both groups, and six gaps in the endocardial group. **Conclusions**: Epicardial ligation of LAA is associated with a longer fluoroscopy time and procedure time compared to the endo approach. Early FUP revealed more thrombi in the epicardial group and more PDLs in the endocardial group. A 1-year FUP and long-term FUP showed comparable clinical results.

## 1. Introduction

Atrial fibrillation (AF) and its thrombogenic effect in the LAA is responsible for a high percentage of cardioembolic strokes [1]. Stroke risk could be mitigated by effective oral anticoagulation (OAC) [2,3]. Concomitant elimination of the LAA during cardiac surgery has demonstrated significant reduction in stroke rate and mortality [4,5]. However, effective long-term anticoagulation is not tolerated by all patients with a high CHADSVASC score due to bleeding complications, repetitive falls, vascular pathologies like Morbus Osler, or amyloid angiopathy. LAA closure has been established as a therapeutic alternative for those patients not tolerating OAC. Its non-inferiority to OAC has been proven by several large randomized trials [6,7,8,9]. Many different devices are available for percutaneous LAA occlusion [10]. Watchman Flex (former Watchman, Boston Scientific, Marlborough, MA, USA) and Amulet (former ACP plug Abbott, St. Paul, MN, USA) are most frequently implanted. Epicardial ligation using the Lariat device (Atricure, Dayton, OH, USA) is unique to other approaches as no device is implanted and anticoagulation can be omitted after procedure. One limitation is the need for epicardial access, its associated challenges and long learning curves, as well as the presence of a department of a cardiac surgery.

In our institution, epicardial ligation is the standard approach for LAA occlusion in patients with no contraindication for epicardial puncture (Table 1). Patients after cardiac surgery, with epicardial adhesions or retropulmonic LAA morphology, were treated endocardially with the Amulet device. Both groups were stratified with a preponed CT imaging.

We analyzed the procedure data of the endocardial LAA occlusion using Amulet and the epicardial ligation with Lariat, and their 1-year follow-up (FUP) data in patients with atrial fibrillation and high risk for stroke and bleeding retrospectively.

## 2. Methods

From November 2018 until August 2024, 112 patients were included in this retrospective analysis. All patients signed for informed consent about the procedure and the scientific evaluation. All patients received a CT scan of their heart to stratify the best approach. Anatomical landmarks, like LAA morphology and size, pericardial thickening, pectus excavates or sternal cerclage after cardiac surgery, were considered. A total of 69 patients (50.7% male) were treated with epicardial ligation using Lariat (epicardial group) and 43 (61.9% male) were treated with endocardial occlusion using Amulet (endocardial group). One patient was treated with both techniques due to a very large LAA and was excluded from this analysis. One patient was successfully treated for neither epicardial (adhesions) nor endocardial ligation (air aspiration due to refractory negative LA pressure). In 38% of cases, contraindication for epicardial ligation was prior cardiac surgery, in 38% it was anatomical considerations, and in 21% pericardial adhesions (mostly due to uremic pericarditis). One patient denied epicardial approach (see Table 1 and Table 2).

### 2.1. Epicardial Procedure (Figure 1)

**Figure 1:**
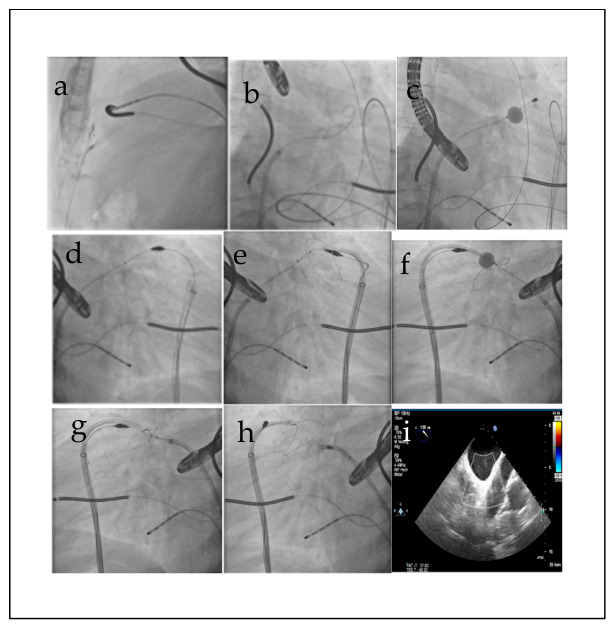
Stepwise approach to the epicardial ligation of the LAA: (**a**) epicardial puncture and insertion of the soft sheet, (**b**) transseptal access with LAA angiography with pigtail catheter, (**c**) endowire position in the anterior lobe of the LAA, (**d**) epiwire position with connection of wire magnets, (**e**) opened snare of the LARIAT device passing the LAA, (**f**) epicardial LARIAT device placed in position over LAA along the epiwire, (**g**) ligation of the LAA using the LARIAT snare device, (**h**) release of the snare after tightening the sutures, and (**i**) final TOE (130°) with documented complete ligation of LAA.

After having given informed consent, the patients were put under analgosedation with continuous propofol infusion combined with boli of piritramide. TOE was inserted and a thrombus in the LAA was ruled out. After puncturing the pericardium and introducing a soft tip in the pericardial space under echocardiographic control of the right ventricle, transseptal puncture is performed under transesophageal echo (TOE) guidance [11]. Identifying the anterior lobe in the TOE combined with the angiogram of the LAA, an endocardial wire with a magnet at its end is placed in the LAA anterior lobe. Via epicardial access, a second magnet-attached wire is introduced and connected to the endocardially placed magnet. Over the connected magnet wires, the snare is advanced over the LAA and positioned at the LAA neck. The optimal closing position is confirmed by angiogram, balloon insufflation and TOE guidance. After closing the snare, complete capture of the LAA is confirmed by TOE. The suture is released from the snare. After two tightenings of the suture with a tension device, the result of the ligation is confirmed by TOE for central gap or missed lobes. The suture is cut and a pigtail drain is placed in the pericardial space (Figure 1). The patient is monitored in the ICU for 24 h. For prevention of pericarditis, treatment with colchicine was started before the procedure and continued for 6 weeks. All patients were discharged without any form of anticoagulation, except the ones with known coronary heart diseases being treated with 100 mg acetyl acid/d.

### 2.2. Endocardial Procedure (Figure 2)

**Figure 2:**
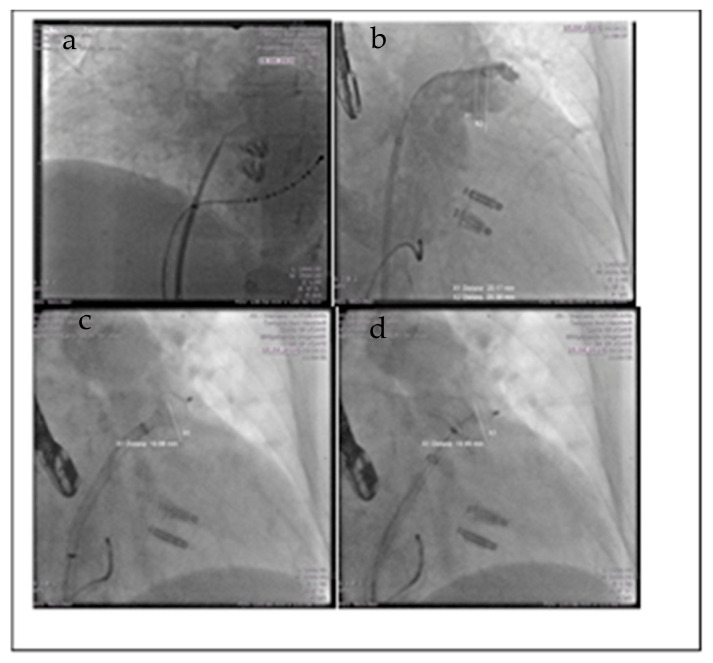
(**a**) Transseptal puncture in LAO 50°, (**b**) angiographic presentation of LAA in RAO 30°/15° cranial, (**c**) implantation of device with release of body, (**d**) after release of disk with correct positioning of whole device.

After having given informed consent, the patients were put under sedation with continuous propofol infusion. TOE was inserted and left atrial thrombus was ruled out. the sizing parameter of the LAA was documented. After puncturing the femoral vein and administering heparin, transseptal puncture was performed under TOE guidance (Figure 2a) and a Lamp sheet was introduced to the left atrium. Intravenous volume guidance was adjusted to the left atrial pressure. Heparin dosage was guided by maintaining an ACT of >250 s. Via pigtail catheter, a biplane cine of the LAA was performed for sizing of the LAA (Figure 2b). After appropriate device size selection based on the echo- as well the angiographic measurements, the sheet system was changed to a 12 or 14 French double-curved device access sheet with an over the wire technique. The device was introduced to the left atrium and advanced under fluoroscopic and TOE-guided control to the LAA. Upon reaching the landing zone, the device is slowly deployed by unsheathing the device under fluoroscopy (Figure 2c). In the proper position, the body of the disk is deployed in a second step. Optimal seal, stability and compression have to be confirmed by TOE and fluoroscopy (Figure 2d). In case of an inadequate result, the device can be resheathed, and the implanting process repeated until an optimal result is achieved. After two tug tests, complete seal of the LAA ostium and sufficient compression, the device is released. Pericardial effusion is excluded for the next 3 days, and dual antiplatelet therapy (DAPT) is initiated after the procedure and continued for 3 months.

All patients with no contraindication for MRI received a brain MRI for detection of periprocedural silent cerebral lesions (SCLs) on the following day within 24 h. SCLs were defined by hyperintensities (bright spots) in MRI diffusion weighted imaging to detect acute brain ischemia. These spots were correlated with hypointensities in the apparent diffusion coefficient mapping to rule out artifacts. The MRI interpretation was blind.

Patients’ data, procedural data and MRI data were documented during the hospitalization. Minor complications were defined as requiring normal therapy or no treatment but longer observation. Major complications impact patients’ care, treatment and resource utilization significantly.

### 2.3. Follow-Up

TOE control and clinical visits were scheduled after 3 months and 12 months for both groups. Thrombus formation (size and location) or leaks detected by a color Doppler (size and location) were documented, as well as clinical events like death or stroke.

In cases of complete seal of the LAA in the endocardial group, DAPT was reduced to 100 mg ASA. In cases of relevant gaps > 5 mm, DAPT proceeded and a second TOE control was performed after 6 months. All patients (in the endo- and epicardial groups) with relevant coronary artery disease were treated with 100 mg ASA.

In case of a thrombus, NOAK was initiated and a TOE control was performed after 6 weeks.

Hospitalizations after 12 months for different medical reasons were screened and documented.

### 2.4. Statistical Analysis

The data are expressed as mean ± standard deviation (SD) for continuous variables or as numbers and percentages for categorical variables. T-test was used for mean values, and Chi-square test for numbers. A *p*-value of 0.05 was considered statistically significant.

## 3. Results

### 3.1. Procedure

The endocardial patient cohort was older; more patients suffered from prior stroke and coronary heart disease (CHD) and had a higher HASBLED score. Successful closure of the LAA was achieved in 98.6% with the epicardial approach (one failure due to adhesions) and in 41 patients (97%) with the endocardial approach (one failure due to refractory air aspiration). In both groups, one major complication occurred: one patient developed a large esophageal hematoma after starting DAPT in the endocardial group; in the epicardial group, one laceration of the LAA occurred requiring surgical closure with Atriclip. Minor complications occurred, with four patients (5.7%) in the epicardial group developing pericarditis. No access site complications were seen. Procedure time (62 min vs. 83 min, *p*< 0.001) and fluoroscopy time (9 min vs. 17 min, *p* < 0.001) were significantly longer in the epicardial group. MRI was performed in 24 patients of the epicardial group and in 25 patients of the endocardial group. Silent cerebral lesions are distributed equally (11% vs. 8%, *p* < 0.67) (Table 3).

### 3.2. 3-Month FUP

TOE showed five thrombi in the epicardial group, no thrombus in the endocardial group. In the endocardial group, six PDLs were described (mean size 4.2 mm, range 8–2 mm) and one central gap was detected in the epicardial group. There was one death in the endocardial group with cerebral bleeding under DAPT and one death with fatal stroke in the epicardial group without any TOE before death. No stroke or systemic embolism was reported.

### 3.3. 12-Month FUP

TOE showed no thrombus either in the epicardial group or in the endocardial group. All thrombi were resolved under NOAK therapy and did not recur within 1 year (one patient with positive Lupus anticoagulants was kept on half NOAC). The central gap in the epicardial group closed within 1 year, and the PDLs in the endocardial group remained unchanged under ASA therapy. No death or stroke/embolic event was reported.

### 3.4. >12-Month FUP

Mean FUP interval for 10 patients in the endocardial group was 15 months ± 33 (range 71–13 months) and for 29 patients in the epicardial group it was 17 months ± 31 (range 60–13 months). Two strokes were reported in both groups; three patients without any findings in the TOE and one patient in the endocardial group with a known PDL of 2 mm were reported. Six patients died in the epicardial group, all non-related to stroke or embolism, and one patient died in the endocardial group. No new development of thrombus or gap could be observed (Table 4).

## 4. Discussion

This retrospective single-center experience study analyzes procedure data and clinical efficacy of two different methods for closing the LAA for stroke prevention in patients with atrial fibrillation and contraindication for DOAC retrospectively. Direct matching of both patient cohorts is not possible as the cohorts are statistically not independent. Although the techniques differ significantly in procedure and fluoroscopy time, the periprocedural silent cerebral lesions were similar between the 2 groups. The epicardial group demonstrated earlier thrombus in patients with spontaneous echo contrast which resolved and did not recur or lead to strokes during the 12-month FUP, whereas the endocardial group was associated with more PDLs which remained patent during the FUP. The long-term FUP showed high efficacy of both methods with similar rates of severe adverse events.

The epicardial LAA ligation is a more complex procedure in regard to anatomy and clinical parameters, explaining the longer procedure duration and the longer radiation time. Epicardial puncture requires a longer learning curve and can be associated with severe complications [12]. In addition, in-house cardiac surgery is a prerequisite for epicardial ligation. In our series, one severe complication with laceration of the LAA was encountered due to failure to deploy the suture completely. This patient was treated with Atriclip after thoracotomy by the cardiac surgeons and recovered well. No complications concerning the epicardial puncture were identified. Endocardial device implantation can also be associated with severe complications. In our cohort, one patient developed a giant hematoma in the esophagus after loading with DAPT, thus requiring intervention. Other complications, like device dislocations, early and late pericardial effusions or even severe air embolism due to the large sheath size, were not observed in our cohort. All patients develop a more or less mild pericarditis after epicardial ligation, despite prophylactic treatment with colchicum. In four patients, the anti-inflammatory therapy had to be escalated to steroids. The challenge of the endocardial approach is the correct sizing and achievement of an optimal closure with no PDLs [13].

PDLs are a relevant issue as their incidence is reported as ranging from 1.8% to 25% depending on definition [14] and may be avoided with an effective LAA occlusion aiming at a high compression of the device [15]. Meanwhile, minor PDLs < 5 mm may also cause stroke or systemic embolism [16,17]. Optimal device fitting and implantation is crucial for the success of the endo-procedure, as the PDLs do not close spontaneously during FUP and can be associated with stroke [17,18]. All six PDLs in our cohort were still detectable after the 1-year FUP and were maintained on single antiplatelet therapy, with one stroke in the FUP (patient was under clopidogrel); in the epicardial group, there was one central gap which closed spontaneously. The incidence of no central gaps in the prospective randomized aMAZE trial was reported to be 84% of patients, and less than or equal to 5 mm residual communication was observed in 99% of patients [19]. In cases of persistent gap, they may be closed by implantation of a plug device [20]. Long-term anticoagulation was Asa in all patients in the endocardial group. In the epicardial group, only the patients with concomitant coronary heart disease were treated with Aspirin (30%). The great advantage of epicardial ligation is the possibility to omit all forms of anticoagulation after the procedure as no foreign body is implanted. Patients with a very high bleeding risk, like in cerebral amyloid vasculopathy and Morbus Osler, especially benefit from omission of anticoagulation.

Of major concern is the high rate of occlusion-related thrombus in the epicardial group revealed during the first 3 months’ FUP: five thrombi (7.2%) in the epicardial group and none in the endocardial group. The incidence of thrombus after epicardial ligation is reported to range from 0.5% [19], 1.5% [21] to 3% [8] in contrast to the endocardial approach with a DRT rate of 3.8% to 7.2% [22,23]. Our patients with thrombus showed in one younger patient coagulopathy and in three female patients high age (≥85 year) combined with spontaneous contrast in the TOE; in one patient, no risk factor for PDLs could be identified. None of these five patients developed a stroke or systemic embolism. All thrombi were resolved by reinitiation of anticoagulation with DOAK, and did not recur during the 12-month FUP. Early TOE control and detection of thrombus along with reinitiating DOAK may be crucial after epicardial ligation.

The incidence of SCLs in a post-procedural brain MRI is comparable in both procedures, with 8% in the endocardial group and 11.5% in the epicardial group. This rate is low compared to the data of Wang et al. [24], with 38% silent cerebral lesions, or the 32% [25] reported by Majunke et al. after endocardial LAA occlusion. A reason for the low number of SCLs might be the careful manipulation of air trapped in the sheets. Whether SCLs are related to air or embolism or both is still unclear.

Clinical events in both groups during long-term FUP were distributed equally: no new thrombus or central gap/PDL was reported. Two strokes occurred in each group. In 3 patients, no findings in the TOE were reported, but in one patient, a known PDL of 2 mm could be detected, confirming the results of various trials mentioned before. All six deaths of the epicardial group during FUP were related to noncardiac causes (COVID infections, sepsis, cancer).

## 5. Limitation

As the endocardial group represents the patients with contraindication for epicardial ligation, no propensity score matching between both groups is possible.

These data derive from a retrospective observational single-center study, with all its limitations due to patient selection, patients’ characteristics and patients’ compliance concerning medication intake and follow-up, as well as selection bias based on preprocedural cardiac CT imaging.

## 6. Conclusions

Acute closure success and safety of the LAA is comparable with both procedures. Both approaches for LAA closure show comparable long-term FUPs concerning clinical events. All forms of anticoagulation may be omitted after epicardial ligation. The longer procedure and radiation time in the epicardial approach may be robustly shortened with future modifications of LAA epicardial ligation techniques using the same device, which is still under investigation.

## Figures and Tables

**Table 1:** Contraindications for epicardial LAA ligation and distribution on our cohort.

Clinical Contraindications (n = Patients)	Anatomical Contraindications (n = Patients)
History of cardiac surgery **16 (38%)**	Backwards orientated LAA with the anterior lobe behind the pulmonary trunk **16 (38%)**
Renal failure with dialysis **7 (10%)**	Left rotated heart
Pectus excavates	LAA width > 50 mm
History of thoracic radiation	Multiple lobes with different orientations and wider distance than 50 mm
NYHA IV classification	Adipositas BMI > 50
Planned cardiac surgery with surgical LAA resection	Thrombus in LAA
Adhesions **2 (3%)**	

**Table 2:** Patients’ characteristics (t-test for mean values, Chi-square test for absolute values).

Patients’ Characteristics	Endocardial Group n = 42	Epicardial Group n = 69	*p*-Value
Gender male	26 (61.9%)	35 (50.7%)	0.326
CHADSVASC score	4.3	4.0	0.271
HAS BLED score	3.8	3.3	0.001
Diabetes	6 (14.3%)	15 (21.7%)	0.333
Coronary heart disease	22 (52.4%)	21 (30.4%)	0.022
Prior stroke	12 (28.6%)	9 (13.2%)	0.048
Age	78.57	74.74	0.012
BMI	26.9	27.9	0.334

**Table 3:** Procedural characteristics; t-test for mean values and Chi-square test for absolute values.

Procedural Characteristics	Endocardial Group	Epicardial Group	Statistical Test
Procedure time min	62 ± 20	83 ± 24	<0.001
Fluoroscopy time min	9 ± 6	17 ± 23	<0.001
Silent cerebral lesions	2 (11%)	3 (8%)	0.67
Minor complication	0	4	
Major complication	1	1	

**Table 4:** Incidences of thrombus, PDL, death and stroke after 3 months, 12 months, and >12 months; Chi-square test for absolute values.

FUP	3 Months			12 Months			>12 Months	
Procedure	Endo	Epi	*p*-Value	Endo	epi	*p*-Value	Endo	epi
Thrombus	0	5	0.08	0	0	1.0	0	0
PDL	6	1	0.13	6	0	0.05	6	0
Death	1	1	1.0	0	0	1.0	1	5
Stroke	0	0	1.0	0	0	1.0	2	2

## Data Availability

The original contributions presented in this study are included in the article. Further inquiries can be directed to the corresponding author.

## Abbreviations

CHD	coronary heart disease
DAPT	dual antiplatelet therapy
DOAK	direct oral anticoagulation
FUP	follow-up
ICU	intensive care unit
LAA	left atrial appendage
PDL	peridevice leak
SCL	silent cerebral lesion
TOE	transesophageal echo
